# Alkaline Activity of Portland Cement with Additives of Waste Glass

**DOI:** 10.3390/ma14061346

**Published:** 2021-03-10

**Authors:** Wiktor Szewczenko, Galyna Kotsay

**Affiliations:** Faculty of Civil Engineering, Mechanics and Petrochemistry, Warsaw University of Technology, 17 Łukasiewicza St., 09-400 Płock, Poland; galyna.kotsay@pw.edu.pl

**Keywords:** waste glass, ordinary Portland cement, alkaline activity, mortar

## Abstract

The concept of the alkaline activity of powdered materials introduced into cement compositions has been proposed, along with methods for its determination. The possibility of using waste glass as an active additive to Portland cement was evaluated from the standpoint of alkaline activity. Replacing the Portland cement component with glass waste in the form of glass powder at amounts from 1 to 35% made it possible to maintain the cement composition’s alkaline activity at a level that met the standard requirements. The previously unknown effects of mixed alkali in Portland cement in the presence of glass waste are described. Portland cement has a high potassium alkaline activity; however, container glass has a high sodium alkaline activity and a fairly low potassium alkaline activity. When glass waste is introduced into the structure of cement compositions, potassium alkaline activity is reduced.

## 1. Introduction

Despite the large amount of waste glass present globally, especially from glass containers, waste glass has not yet been used as an active additive in Portland cement. Waste glass is not listed as a main component of ordinary cement following the standard [1]. At the same time, though, metallurgical slags containing a relatively large amount of glass phases are among the active additives mentioned in the standard. Over time, glass waste may also be included as an active additive in cement.

In previous works [2,3,4,5,6,7,8], waste glass was shown to be the best addition to cement, because glass has a high content of amorphous silica. Fine-ground waste glass with particle sizes below 100 µm was proposed as a pozzolanic material. In [9], it was proven that for powdered waste glass with sizes smaller than 40 μm, the compressive strength index reached more than 82%. According to [10,11,12,13,14,15], fine-ground glass powders have great potential as a partial replacement for cement in mortar and concrete. Glass powder with a Blaine specific surface area higher than 250 m^2^/kg exhibited very high pozzolanic activity. Additionally, 30% of ground waste glass with sizes from 45–75 µm could be incorporated as cement replacement in mortar or concrete without any detrimental effects caused by alkaline corrosion. Ref. [16] proposed using recycled waste glass to replace 10% to 60% of cement. The mortars containing 60% recycled waste glass and superplasticizer were able to achieve 99 MPa, compared to the strength of 115 MPa achieved by the plain cement sample. Compared to silica fume and fly ash, glass has higher amounts of Na_2_O and K_2_O, resulting in alkaline corrosion and excessive expansion in concrete. Recent studies have shown that the particle size of glass is a crucial factor for alkali-silica reactions. According to [17,18], when the waste glass is finely ground to below 75 µm, the alkali-silica reaction expansion does not occur, and mortar durability is guaranteed.

The effects of waste glass as an additive or aggregate on the mechanical properties and durability of concrete are not conclusive [19,20,21,22,23]. Researchers have studied new alternative binders with alkali-activated materials or polymeric glass composites [24,25,26,27,28,29,30]. Considering the limitations on the alkali content in ordinary concrete, it is necessary to control the alkaline activity of the glass waste, which may fluctuate significantly. In connection with the above, the purpose of this work was to develop a method for controlling the alkaline activity of glass waste and its impact on the alkaline activity of cement–glass compositions. 

## 2. Materials and Methods 

The objects of the study were powdered waste glass with a grain size <0.063 mm and ordinary Portland cement: CEM I 32.5 R, CEM I 42.5 R, CEM I 42.5 N produced by Group Cement Ożarów (Ożarów, Poland). Chemical analysis of the cement was conducted following the standard [31], and that for glass and silica fume was carried out in accordance with [32]. The chemical compositions of the materials are shown in Table 1.

For the quantitative analysis of alkali content, a flame photometer FP902 (PG Instruments Limited, Alma Park, Wibtoft Leicestershire, UK) with an accuracy of ±0.5% was used. The materials’ alkaline activity was determined on the basis of the content of sodium and potassium cations in the solution after extraction from 1 g of material in 100 mL of distilled water during 30 s exposure. The alkaline activity of the powder materials is presented in units of ppm/g and ppm/m^2^ of material. The specific surface measurements of the materials were conducted by Blaine’s flow method according to [33]. To determine cement products’ alkaline activity, distilled water was used as an extractant with a ratio of sample surface to extractant volume of 0.34 cm^−1^. The samples were prepared from CEM I 32.5R (450 g), standard sand (1350 g) and distilled water (225 g). When preparing the cement compositions, the dry cement was mixed with the appropriate amount of glass powder for 3 min; then, the mixture was poured into water and sand was added while mixing. Each mortar was cast in molds of 40 mm × 40 mm × 160 mm. All cuboids of mortars were taken out from the molds after one day, and the alkaline activity of the mortar was then determined. The results of alkaline activity of molded product are presented in units of ppm/m^2^ of mortar.

## 3. Results

It is known that the activity of sodium and potassium ions in cement depend on whether they occur as sulphates or form solid solutions in clinker phases. The sodium and potassium occurring in solid solutions are slowly released into solution, while ions occurring in sulphates are rapidly removed. However, the alkaline activity also depends on whether alkaline cations are bound inside or on the material’s surface. Alkaline cations are in a nonequilibrium state on the surface of the grains. The activation energy of these leaching cations is lower than that of the cations inside the material [34]. This suggests that such cations are active, and can be easily extracted under appropriate conditions. Accordingly, the number of cations extracted from a surface unit per unit of time is called the cement’s alkaline activity and its additives. We previously used a similar definition only for glass powders [35,36]. 

### 3.1. Alkaline Activity of Materials Used in Cement Compositions

The alkaline activity was determined for ordinary Portland cement and additives: container glasses, borosilicate glasses, silica fume. Table 2 represents the alkaline activity results of the investigated materials. 

Portland cement has a high potassium alkaline activity, which exceeds sodium by more than one order of magnitude. If the alkaline activity of Na^+^ for different types of cement varies within 10%, then this variation is more than two times higher for K^+^. An opposite situation is observed for container glass: a high sodium alkaline activity and relatively low potassium activity. It should be noted that the alkaline activity of glass powders is proportionally dependent on the alkali oxide content in the glasses. Additionally, the low sodium alkaline activity of silica fume should be noted, although potassium’s alkaline activity is close to the alkaline activity of container glass.

**Figure 1:**
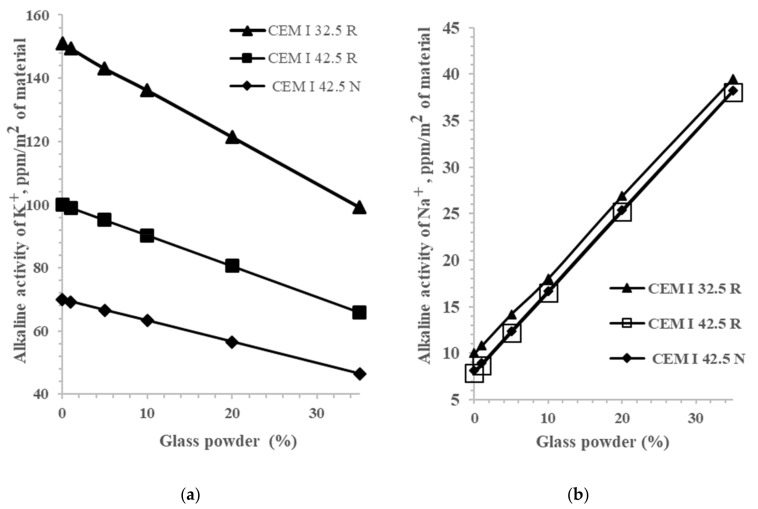
The alkaline activity K^+^ (**a**) and Na^+^ (**b**) of various cement types with varying mass replacement of waste glass powder.

Considering that brown glass has the highest alkaline activity of Na^+^, the next tests were carried out for systems in which glass powder was introduced instead of cement. Using the specific alkaline activity values for each material’s sodium and potassium cations, the alkaline activity for cement compositions was calculated according to [36]. The results of the alkaline activity of sodium and potassium are shown in Figure 1.

As presented in Figure 1, when we replaced various cement with glass powder in an amount of 1–35% (35%, i.e., a higher acceptable limit of the additive content according to [1]), the alkaline activity of K^+^ decreased; however, the alkaline activity of sodium increased. The total sodium and potassium cations’ total activity decreased for all cement compositions and did not exceed the permissible level of 161 ppm, which corresponded to the complete alkali content of 0.6% (Table 3).

Increasing the glass content decreases the overall alkalinity for cement compositions CEM 32.5 R and CEM 42.5 R; however, it increases for systems with cement CEM I 42.5 N. When glass powder additives were introduced above 100% (Table 4), a permissible value of alkaline activity was achieved, with the total alkali content already being at 1%. At an additive content of 35%, the alkaline activity exceeded the permissible value by 21%. These data show that the cement’s intrinsic potassium alkaline activity plays a crucial role in the total alkaline activity.

### 3.2. Alkaline Activity of Cement Products with the Addition of Glass Powders

**Figure 2:**
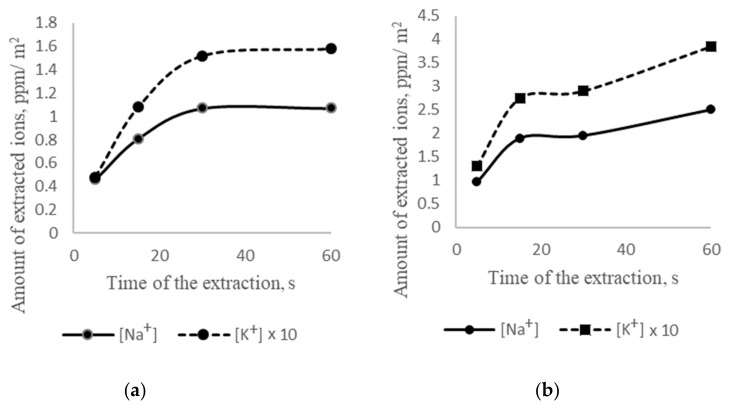
The amount of extracted Na^+^ and K^+^ vs. temperature and time of the extraction process from the mortar’s surface layers: (**a**) 295 K and (**b**) 368 K.

It is known that the amount of extracted alkaline cations is dependent on the temperature and time (exposure) of the extraction process [37]. Accordingly, the effect of temperature and time on cement product extraction was studied (Figure 2a,b). The extraction was made on cuboids of mortars at temperatures 295 K and 368 K.

According to Figure 2a, it can be argued that at 295 K, the amount of extracted cations increased with the exposure from 5 to 30 s and further stabilized. Thus, the exposure time of 30 s may be considered the optimal exposure time. The number of extracted potassium cations was 15 times higher than that of extracted sodium cations, as in cement powder. At 368 K, the extraction process was two times faster than that at 295 K. The stabilization process occurred after 15 s; however, after 30 s, the amount of extracted cations increased again (Figure 2b).

Thus, the temperature was an essential factor for increasing the number of extracted cations per time unit. However, the extraction at 368 K has a significant drawback due to uneven heating of the sample in such a short time. Accordingly, it was proposed to carry out the extraction process at room temperature with high-frequency electromagnetic radiation (HER), which allowed an intensification of the leaching process (Table 5). 

**Figure 3:**
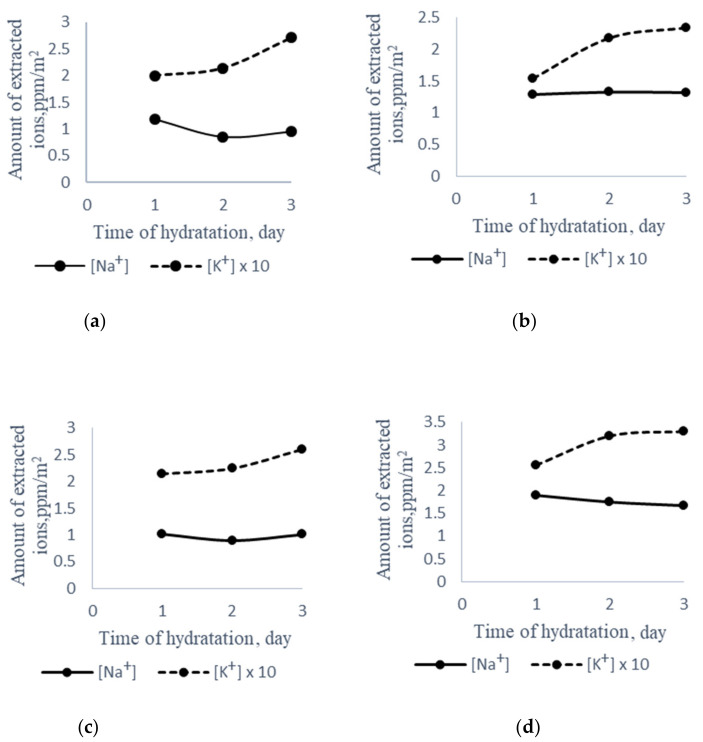
The amount of extracted ions from surface layers of the mortar vs. time of hydration of the cement: (**a**) 100% CEMI 32.5 R, (**b**) 90% CEMI 32.5 R + 10% colorless glass, (**c**) 90% CEMI 32.5 R + 10% borosilicate glass, (**d**) 90% CEMI 32.5 R + 10% silica fume.

The extraction process with electromagnetic radiation made it possible to almost double the number of extracted cations without increasing the extractant’s temperature. Considering that the structural matrix of cement stone changes during the period of hydration, we aimed to determine the influence of such structural changes on the alkaline extraction process from mortar cement without additives and from mortars cement with glass powders having different alkaline contents and silica fume (Figure 3). 

As presented in Figure 3a, an increase in alkaline potassium activity of 33% was observed for mortar made from ordinary Portland cement after three hydration days. Simultaneously, sodium alkaline activity decreased by 23%. The introduction of colorless glass powder reduced the potassium alkaline activity by 25%; however, the tendency of growth with the increase in hydration time was the same result as for pure cement (Figure 3b). A similar situation was observed for borosilicate glass (Figure 3c). For silica fume (Figure 3d), the potassium alkaline activity increased in comparison with other additives, which is most likely due to the high specific potassium alkaline activity of silica fume (see Table 2).

For glass powders, it was found that the first extraction was the most effective [37]. More than 90% of alkali content was removed from the surface. When using two and three extractions, the amount of extractable cations was reduced to 2–6%. Relevant studies were conducted to determine whether this trend persists for Portland cement, and the results are presented in Table 6.

Table 6 shows that the amount of extracted K^+^ in all compositions increased by 35–40% during the second extraction. However, the third extraction showed a sharp decrease in this value (by 75%). The data indicate that during the day after the first extraction, the pore fluid enriched with potassium ions still migrated to the sample surface. The partial blockage of the pores caused a sharp decrease in the number of extracted ions during the third extraction. The reasons for such blockage will be discussed further. When 10% of glass powder was added to cement, there was a decrease in extracted potassium cations regardless of the number of extractions. This may indicate the suppression of potassium alkaline activity in Portland cement with the high sodium alkaline activity of glass powder, which allows us to predicate the phenomenon of the mixed alkaline effect on the porous structure of Portland cement, in contrast to the mixed alkaline effect in glasses with a dense, nonporous structure.

When glass powder was added to the cement, its surface alkaline cations instantly passed into the liquid phase, increasing its alkali content. Then, the surface layer of the glass grains was dissolved under the influence of the alkaline medium of the pore fluid. The partial dissolution of the glass resulted in the formation of potassium and sodium metasilicates in the pore fluid according to the following reaction:

2 ROH + SiO_2_ → R_2_SiO_3_ + H_2_O (1)

Furthermore, with an excess of H_2_O, the following reaction occurred:

R_2_SiO_3_ + m H_2_O → 2ROH + 2 H_2_SiO_3_ (2)

H_2_SiO_3_ → SiO_2_ (gel) + nH_2_O (3)

(where R^+^—Na^+^ or K^+^).

The SiO_2_ gel contained in the pore fluid adhered to the pore walls and reduced their diameter. In this way, it blocked the movement of potassium cations to the cement product’s surface, as the potassium cations had a larger ionic radius compared to the ionic radius of the sodium cations. The decrease in the potassium alkaline activity in the pore fluid occurred due to the high sodium alkaline activity of the glass powder, which is referred to as the mixed alkaline effect in cement.

## 4. Conclusions

Replacing the Portland cement clinker component with glass waste in the form of glass powder at amounts from 1 to 35% made it possible to maintain the cement composition’s alkaline activity at a level that meets the standard requirements. Simultaneously, the introduction of the same amount of glass powders above 100% exceeded these requirements, even when introducing only 1% of powder. These results indicate that the main role played by alkali content is in the potassium alkaline activity of Portland cement.

In addition, this article proposed carrying out the extraction process at room temperature with high-frequency electromagnetic radiation, for intensification of the leaching process. 

Based on the research performed, it can be concluded that the addition of glass waste in the form of a powder contributes to the increase in the sodium alkaline activity of cement products, which are characterized by high potassium alkaline activity. Because of the glass powder, an unknown phenomenon was observed with respect to the mixed alkali effect in Portland cement. When 10% of glass powder was added to the cement, there was a decrease in the extracted potassium cations, regardless of the number of extractions. This may indicate the suppression of potassium alkaline activity in Portland cement with the high sodium alkaline activity of glass powder, which allows us to preicate the mixed alkaline effect phenomenon on the porous structure of Portland cement. When glass waste is introduced into the structure of cement compositions in large quantities, it is necessary to consider the reaction of the glass grains with calcium hydroxide during long-term exploitations.

## Figures and Tables

**Table 1:** Chemical composition of materials (wt.%).

Oxides	Additives					Ordinary Portland Cement		
	Container Glasses			Borosilicate Glass	Silica Fume	CEM I 32.5R	CEM I 42.5R	CEM I 42.5N
	Colourless	Brown	Green					
SiO_2_	72.20	72.15	71.80	81.00	95.06	21.74	21.80	21.26
Al_2_O_3_	1.80	1.75	1.80	2.00	1.50	5.00	4.94	4.13
CaO	10.10	10.00	10.97	0.50	0.89	64.74	64.61	64.21
MgO	1.65	1.55	1.00	-	0.45	2.20	2.18	1.88
Na_2_O	13.19	13.79	12.72	4.50	0.25	0.14	0.13	0.13
K_2_O	0.61	0.16	0.63	-	0.42	0.88	0.86	0.47
Fe_2_O_3_	0.04	0.25	0.45	-	0.20	2.30	2.30	5.40
Cr_2_O_3_	0.01	0.03	0.25	-	0.11	-	-	-
B_2_O_3_	-	-	-	12.00	-	-	-	-
SO_3_	0.40	0.32	0.38	-	1.12	3.00	3.18	2.52

**Table 2:** The alkaline activity of materials used for the production of cement products.

Entry	Initial Material	Content in Extractant, ppm/g		Specific Surface, m^2^/g	Specific Alkaline Activity, ppm/m^2^	
		Na^+^	K^+^		Na^+^	K^+^
1	CEM I 32.5R	3.60	54.40	0.36	10.0	151.0
2	CEM I 42.5R	3.10	39.00	0.39	7.9	100.0
3	CEM I 42.5N	3.10	26.60	0.38	8.1	70.0
4	Container glass colourless	26.80	1.16	0.32	83.7	3.6
5	Container glass brown	30.20	1.01	0.32	94.4	3.1
6	Container glass green	20.70	0.86	0.32	64.7	2.7
7	Borosilicate glass	7.40	2.60	0.32	23.1	8.1
8	Silica fume	17.70	41.10	>20.0	0.9	2.0

**Table 3:** Calculated alkaline activity of cement compositions with the addition of brown glass powder.

Glass Powder Replacement (%)	Specific Alkaline Activity Ʃ = Na^+^ + K^+^, ppm/m^2^		
	CEM I 32.5 R	CEM I 42.5 R	CEM I 42.5 N
0	161.0	107.9	78.1
1	160.3	107.7	78.2
5	157.3	107.3	79.0
10	154.6	106.8	80.0
20	148.3	103.1	82.0
35	138.6	104.1	84.7

**Table 4:** The calculated alkaline activity of cement compositions with the addition of brown glass powder above 100% of cement CEM I 32.5 R.

Entry	Glass Powder Introduced, %	Alkaline Activity, ppm/m^2^		The Total Amount of Extracted Cations, ppm/ m^2^
		Na^+^	K^+^	
1	1	10.9	151.0	161.9
2	5	14.7	151.1	165.8
3	10	19.4	151.3	170.7
4	20	28.9	151.6	180.5
5	35	43.0	152.0	195.0

**Table 5:** The effect of extraction conditions on the amount of extracted cations.

Extraction Conditions			Amount of Extracted Cations from the Surface Layers of the Mortar, ppm/m^2^	
Time, s	Temperature, K	HER, MHz	Na^+^	K^+^
30	298	-	0.68	10.90
30	368	-	1.79	28.10
30	298	2450	1.19	20.40

**Table 6:** The effect of the extraction number on the amount of extracted alkaline ions.

N	Composition	Amount of Extracted Alkaline Ions, ppm/m^2^					
		1 Extraction		2 Extraction		3 Extraction	
		Na^+^	K^+^	Na^+^	K^+^	Na^+^	K^+^
1	CEM I 32.5R-100%	1.19	20.40	1.01	27.39	0.50	8.40
2	90% CEM I 32.5R + 10% colorless glass	1.28	15.46	1.60	26.56	0.69	7.48
3	90% CEM I 32.5R + 10% borosilicate glass	1.33	21.50	1.01	26.13	0.54	7.65
4	90% CEM I 32.5R + 10% silica fume	1.90	25.62	1.98	36.30	1.24	17.31

## Data Availability

Data available in a publicly accessible repository.
